# Torticolis musculaire congénital chez l'enfant

**DOI:** 10.11604/pamj.2014.18.190.4863

**Published:** 2014-07-04

**Authors:** Wassia Kessomtini, Wafa Chebbi

**Affiliations:** 1 Unité de Médecine Physique, CHU Taher Sfar Mahdia, 5100 Mahdia, Tunisie; 2 Service de Médecine Interne, CHU Taher Sfar Mahdia, 5100 Mahdia, Tunisie

**Keywords:** Torticolis musculaire, déformation néonatale, muscle sterno-cléido-mastoïdie, muscular torticollis, neonatal deformation, sternocleidomastoid muscle

## Image en médecine

Le torticolis musculaire congénital est la troisième déformation néonatale en terme de fréquence après la dysplasie des hanches et le pied bot varus équin. Il se définit comme une attitude asymétrique et permanente de la tête et du cou par rapport au plan des épaules. Il est dû à une rétraction unilatérale du muscle sterno-cléido-mastoïdien dont l ‘anatomie complexe explique parfaitement la déviation en inclinaison homolatérale, en translation et en rotation controlatérales. Le torticolis congénital est une maladie de nouveau né dont l ‘évolution spontanée est favorable dans plus de 80% des cas. Sa persistance au delà de 4 ans est rare. Nous rapportons l ‘observation d ‘une fille âgée de 4 ans et 6 mois, adressée à l ‘unité de Médecine Physique pour déformation de la tête et du cou. L ‘examen révélait un torticolis droit, une limitation de l ‘inclinaison gauche et de la rotation droite, une contracture du muscle sterno-cléido-mastoïdien droit, un décalage de la hauteur des épaules et des oreilles avec un discret enfoncement de l ‘arcade sourcilière droite et une déviation du menton du nez. Les examens ORL, ophtalmique et neurologique étaient sans particularités. Les radiographies du rachis cervical, des hanches et des membres inférieurs étaient normales. L ‘IRM médullaire, réalisée à la recherche d ‘une malformation de la charnière occipito rachidienne, était sans anomalies. Le diagnostic d ‘un torticolis congénital était retenu. Une ténotomie du muscle sterno-cléido-mastoïdien droit était réalisée, suivie d ‘une immobilisation par une minerve cervicale pendant 3 mois et d ‘une rééducation adaptée. L ‘évolution était favorable avec une restitution complète des mouvements de la tête et du cou et une disparition de la raideur de la nuque après un recul de 3 ans.

**Figure 1:**
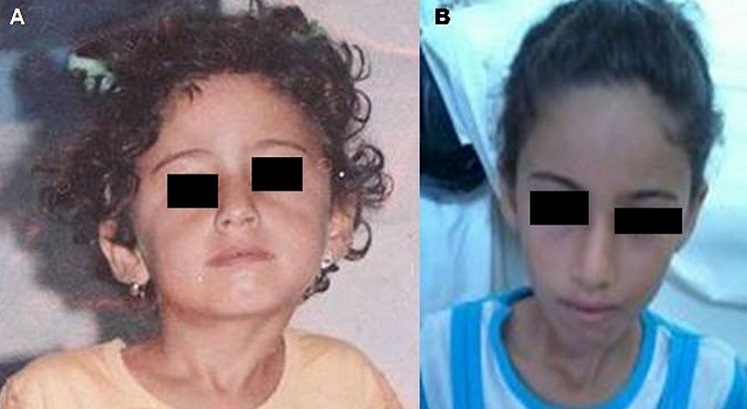
(A) Photo préopératoire: torticolis droit, décalage des épaules et des oreilles, dysmorphie faciale avec un enfoncement de l'arcade sourcilier droit et une déviation du menton du nez; (B) Trois ans après ténotomie du muscle sterno-cléido-mastoïdien droit et rééducation: disparition de la déformation de la tète et du cou

